# Unique Collagen Fibers for Biomedical Applications

**DOI:** 10.3390/md16040102

**Published:** 2018-03-23

**Authors:** Dafna Benayahu, Mirit Sharabi, Leslie Pomeraniec, Lama Awad, Rami Haj-Ali, Yehuda Benayahu

**Affiliations:** 1 Department of Cell and Developmental Biology, Sackler School of Medicine, Tel Aviv University, Tel Aviv 69978, Israel; leslieyael.p@gmail.com (L.P.); lamaawad@mail.tau.ac.il (L.A.); 2 The Fleischman Faculty of Engineering, Tel Aviv University, Tel Aviv 69978, Israel; miritsharabi@gmail.com (M.S.); rami98@tau.ac.il (R.H.-A.); 3 School of Zoology, George S. Wise Faculty of Life Sciences, Tel Aviv University, Tel Aviv 69978, Israel

**Keywords:** marine biomaterials, medical device, scaffold, soft corals, tissue regeneration

## Abstract

The challenge to develop grafts for tissue regeneration lies in the need to obtain a scaffold that will promote cell growth in order to form new tissue at a trauma-damaged site. Scaffolds also need to provide compatible mechanical properties that will support the new tissue and facilitate the desired physiological activity. Here, we used natural materials to develop a bio-composite made of unique collagen embedded in an alginate hydrogel material. The collagen fibers used to create the building blocks exhibited a unique hyper-elastic behavior similar to that of natural human tissue. The prominent mechanical properties, along with the support of cell adhesion affects cell shape and supports their proliferation, consequently facilitating the formation of a new tissue-like structure. The current study elaborates on these unique collagen fibers, focusing on their structure and biocompatibility, in an in vitro model. The findings suggest it as a highly appropriate material for biomedical applications. The promising in vitro results indicate that the distinctive collagen fibers could serve as a scaffold that can be adapted for tissue regeneration, in support of healing processes, along with maintaining tissue mechanical properties for the new regenerate tissue formation.

## 1. Introduction

Acute and chronic injury can cause temporary or permanent damage to tissue. When the body’s natural tissue repair mechanisms are inefficient there is a need to facilitate tissue regeneration. Autologous grafts are limited by the patient’s own tissue and also involve additional surgical procedures. Allografts and xenografts are hard to acquire and require a high compatibility in order to avoid transplant rejection by the recipient as an immune response to the foreign body [1,2,3,4]. There is a growing demand for alternative and improved biomedical devices that are able not only to replace the damaged tissue but also to enable its regeneration and the bio-integration of the implant. Cell function during regeneration relies on the scaffold in order to form new tissue to produce the extracellular matrix (ECM) that will support the cells’ neo-tissue formation. The ECM plays a pivotal role during tissue regeneration, by directing the cell arrangement and modifying their function. Collagen is the most abundant structural protein in the ECM and defines the three-dimensional (3D) structure and biomechanical properties of tissues. 

The challenges in the development of grafts lie not only in promoting cell proliferation and differentiation, but also in providing the grafts with compatible mechanical properties to support the new tissue and facilitate the desired physiological activity [5]. This represents a continuous challenge for researchers and much effort has been focused on developing new materials for tissue repair. Scaffolds can be manufactured from synthetic polymers or natural products [6,7,8]. The advantage of synthetic materials derives from their reproducibility and ability to tailor their physical properties and degradation rate. The degradation rate and porosity allow the tailoring of their use to specific applications, while their limitation is that they cannot be absorbed or integrated with the host tissue, and frequently trigger an immune response [9]. Natural materials that possess a 3D structure are bio-compatible with cells, resulting in better cell attachment and proliferation and consequently facilitating the formation of new tissue and improving regeneration and healing processes [9,10,11,12,13,14,15,16]. The main drawbacks of such materials may relate to their poor mechanical properties or their potential to elicit an immuno-pathological reaction when derived from other mammalian sources (such as bovine, pig, or rat). The use of natural biodegradable bio-polymers for scaffold engineering has several advantages over synthetic materials, such as their similarity to biological macro-molecules, which minimizes immunological reactions and chronic inflammation [5,17,18]. In addition, the natural structures of biomaterials (biopolymers) are compatible and possess properties that support cell attachment. Marine source for bio-polymers such as chitin [14,15], silicified collagen [19,20,21], collagen [12,16,22], or mineralized skeletons [11,13] have been purified from marine organisms such as sponges, jellyfish, and corals and investigated for tissue engineering applications [10]. Corals have also been studied previously as biomaterials for tissue engineering, especially bone tissue engineering, but those studies were mainly focused on mineralized biomaterials [10,11,19,20,21,23]. Here we present a study on unique collagen fibers isolated from the soft coral *Sarcophyton ehrenbergi*, which reveal a 3D structure and superior mechanical properties. The collagen fibers reported were used to create bio-composite materials that demonstrated a unique hyper-elastic behavior similar to that of natural human tissues [24,25,26]. The prominent mechanical properties make these collagen fibers a highly suitable material for biomedical applications. In addition the collagen fibers are endowed by sequence motifs that facilitate and encourage cell adhesion. The current study elaborates upon these collagen fibers embedded with a hydrogel matrix, and these composites were analyzed for their mechanical behavior and biocompatibility in an in vitro model.

## 2. Results and Discussion

**Figure 1:**
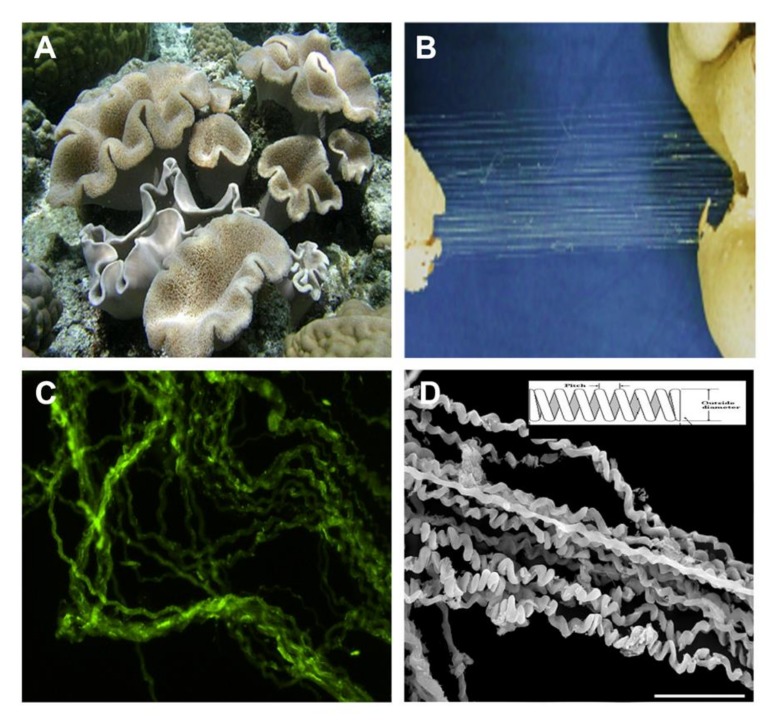
(**A**) Macro image of *Sarcophyton* and (**B**) its torn-apart polypary, revealing collagen fibers pulling from these sections; (**C**) Auto-fluorescence of collagen fibers observed by fluorescence microscopy; (**D**) E-SEM micrographs of collagen fibers that feature a coiled structure.

A recent study on the soft coral *Sarcophyton ehrenbergi* (Figure 1A) revealed ultra-long collagen fibers. These fibers when mechanically pulled from the soft-coral colony (torn parts, Figure 1B) retain their natural physical properties and structure, as opposed to collagen harvested by other methods that destroy its natural structure [6]. These are cord-like fibers that when detached from the mesenteries of the soft-coral tissue reveal a coiled and wavy feature (Figure 1C,D). In earlier studies we revealed the microanatomy of *Sarcophyton auritum* and *Sarcophyton ehrenbergi* colonies, in which the mesoglea between the polyps was shown to contain sparse and short collagen fibers following Masson Trichrome histological staining [27,28]. Additionally, the histological sections revealed eight mesenteries radiating from the inner polyp body-wall across the gastro-vascular cavity and connecting to the pharynx, which contain a mass of collagen fibers. Based on protein MS/MS analysis, these fibers were identified as collagen [27,28].

The fibers are produced and maintained as cord-like bundles in the soft-coral mesenteries. Under a light microscope they demonstrate an undulating appearance. Under fluorescence microscopy an intrinsic auto-fluorescence was detected in the range of 305–450 nm, which is typical for collagen (Figure 1C). Scanning electron microscopy images of these long fibers revealed a coiled spring-like organization (Figure 1D). The coiling presented a pitch-range of 6–40 µm, most probably related to the force applied during their extraction from the polyps. The diameter of the fibers averaged 8.70 ± 1.27 µm (*n* = 57). Such a natural arrangement of the fibers indicates their potential elasticity and suggests that they could serve as a biomaterial with potential for scaffolding applications. The use of high-resolution imaging of scanning and transmission electron microscopy analysis confirmed the striated arrangement and revealed the prominent fibrillary collagenous structure of these fibers [28]. The collagenous type was also confirmed by X-ray diffraction analysis [27].

**Figure 2:**
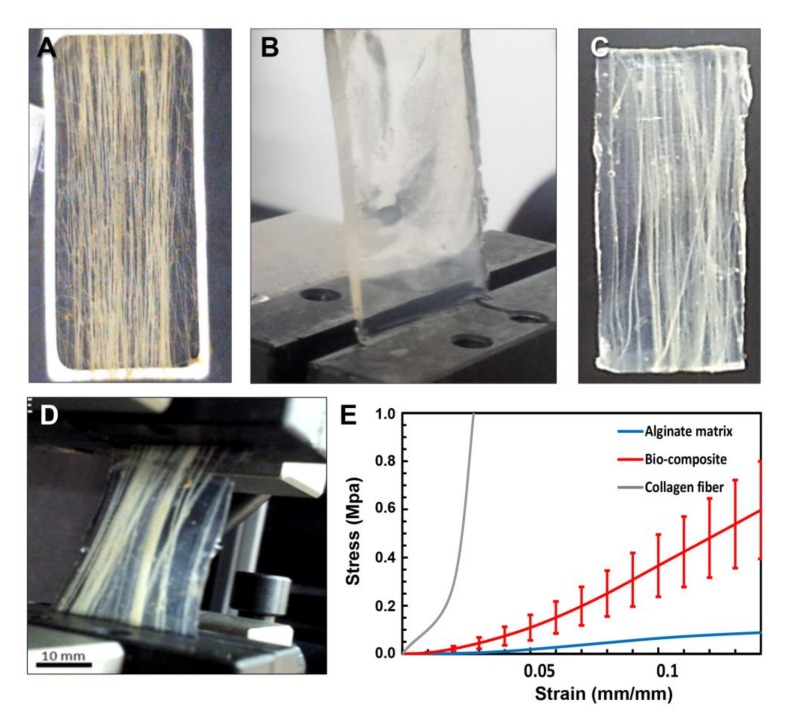
Bio-composite fabrication and mechanical behavior. (**A**) *Sarcophyton* collagen fibers aligned on a metal frame; (**B**) 3% Alginate hydrogel; (**C**) Fabricated uniaxial bio-composite; (**D**) Uniaxial bio-composite under tensile test; (**E**) Mechanical behavior of collagen fibers, alginate matrix, and uniaxial bio-composite. The toe, heel, and linear regions are demonstrated on the *Sarcophyton* collagen fiber stress-strain curve.

**Figure 3:**
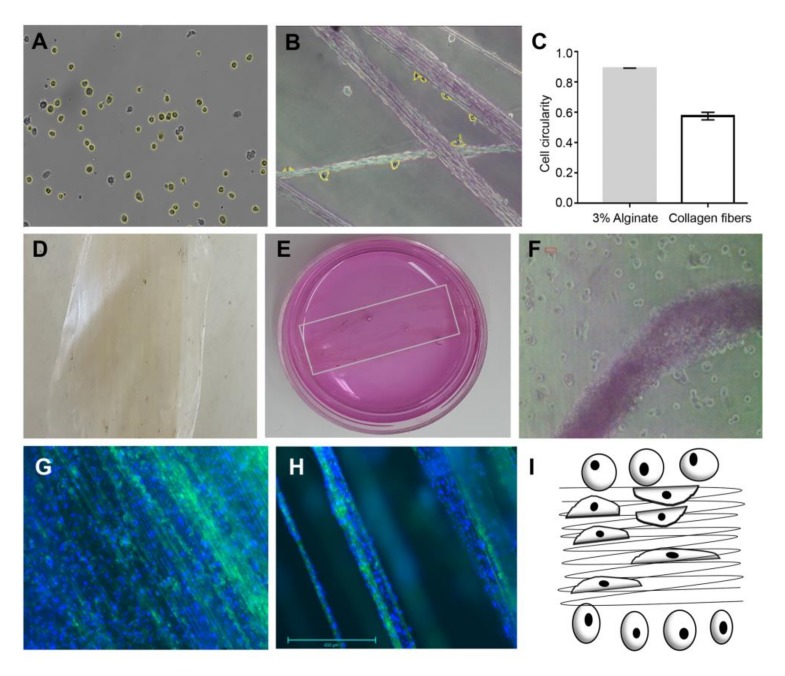
Live imaging and quantification for cells seeded on 3% hydrogel alginate (**A**) on *Sarcophyton* collagen fibers; (**B**) 24 h after seeding cells. Images are at ×100 magnification. (**C**) Cell circularity was analyzed by ImageJ software. Bio-composite was made of collagen fibers embedded in 3% hydrogel alginate. (**D**,**E**) Phase microscopic images of cells seeded on the bio-composite in a tissue culture dish (**F**), were observed to have a mixed morphology of cells: elongated cells are visualized on the collagen fibers along their orientation. On alginate the cells maintained a rounded shape. (**G**,**H**) Cells grown on collagen fibers up to nine weeks formed a tissue-like structure (collagen fibers are shown in green and cell nuclei are DAPI-stained blue). (**I**) Model of substrate stiffness and cells’ morphology. Cells on the fibers are attached and become elongated with the substrate orientation, while cells on the alginate are rounded as a result of no adhesion and the pressure applied by the alginate matrix.

**Figure 4:**
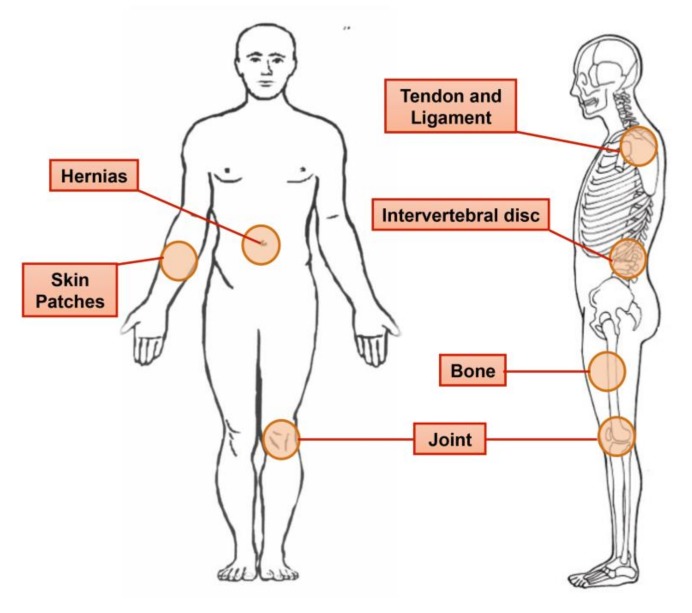
Illustration of potential use of collagen fibers embedded in a hydrogel bio-composite for medical devices with adjusted mechanical properties that provide support and allow motion and flexibility of the tissue under repair.

For potential use of the cord-like fibers for scaffold applications, their mechanical properties were measured (Figure 2) and they were analyzed for cell growth support (Figure 3 and Figure 4). The collagen fibers were pulled out from the soft coral and aligned on a frame (as demonstrated in Figure 2A). In order to create a composite material, the aligned fibers were embedded in an alginate solution (a biocompatible polysaccharide) (Figure 2B) [24,25,26], then immersed in a calcium solution that created ionic bridges and cross-link the alginate hydrogel to create a collagen-alginate bio-composite (Figure 2C). The alginate hydrogel binds the collagen fibers together, creating both an aqueous surrounding and mechanical integrity [26], similar to the mechanical function of proteoglycans in the extracellular matrix (ECM) [29]. The high content of alginic acid with the addition of calcium ions become hydrogel that mechanically resembles the ECM structure of the tissue. The uniqueness of the collagen fibers is manifested in their macro-fibrous structure, ultra-long length, simple isolation, and superior mechanical properties which are rare for a non-mineralized bio-polymer.

### Biomechanics of the Collagen Fibers and Collagen-Alginate Bio-Composites

The collagen fibers were analyzed by differential scanning calorimetry (DSC) to determine the melting point of 68 °C [30], suggesting that these fibers are naturally cross-linked and providing a possible explanation for their exceptional mechanical properties [14,16]. A detailed mechanical analysis of the collagen fibers revealed a non-linear and hyper-elastic mechanical behavior with large deformations similar to that of collagenous soft tissues [31]. The stress-strain curve shown in Figure 2E can be divided into three main regions as reported in the literature for soft tissues [32]: (1) toe region, (2) heel region, and (3) linear region. These regions are also presented for the collagen fiber stress-strain curve in Figure 2E. In the toe region only, a low stress is necessary to achieve a large deformation. This region is a result of the 3D coiling of the fibers (Figure 1D) where the collagen fiber coiling becomes straightened [32]. In the heel region there is non-linear stiffening in the stress-strain curve (Figure 2E), in which the load increases and the collagen fibers straighten with the load direction [32]. The synchrotron by X-ray revealed that a lateral arrangement of the collagen fibrils increased linearly with the strain [33]. The linear region follows the heel region, in which the stress-strain relation is linear, the molecular crimps disappear, and the collagen fibers become straighter at high tensile stresses. The collagen fibers are aligned together in the load direction, becoming straight and strongly resistant to loading, which makes the tissue stiffer and the stress-strain relation returns to linear. Beyond the third phase the ultimate tensile strength is reached and fibers begin to break until tissue failure [32]. For the collagen fibers, the ultimate tensile strength (UTS) was 39–59 MPa (for ~15% strain) and the Young’s modulus in the linear region was 0.34–0.54 GPa [30]. The 3% alginate hydrogel also demonstrated non-linear behavior (*n* = 12), with high deformations, a tensile modulus of 0.91 ± 0.26 MPa and an ultimate tensile strength of 0.19 ± 0.05 MPa, and a failure strain of 0.29 ± 0.08, three orders of magnitude less than that of coral collagen fibers (Figure 2B,E). The collagen fibers retained their coiled structure through post-processing and alignment inside the alginate matrix. The collagen/alginate bio-composite (*n* = 8) with a fiber fraction of 0.33 ± 0.09 demonstrated a similar hyper-elastic behavior to that of the collagen fibers with a UTS of 0.77 ± 0.21 MPa, tensile modulus of 5.54 ± 1.89 MPa, and failure strain of 0.181 ± 0.024 (Figure 2C–E). The alginate hydrogel was found to influence the toe region mechanical behavior of the bio-composite stress-strain curve, while the collagen fibers created the stiffening effect at the heel and linear regions [24,25].

The bio-composite material enables the production of 3D structures that allow cell growth while retaining the natural mechanical features of the fibers. In the literature, some composite materials have been presented based on synthetic electro-spun fibers such as poly lactic-co-glycolic acid (PLGA) or on natural materials such as jellyfish collagen [12,18]. The hybrid scaffold demonstrates an effective mechanical stability and biocompatibility. Considering its marine origin and the evolutionary distance from vertebrates, this minimizes the risk normally associated with a mammalian collagen [12]. The hybrid combination affects the biocompatibility, proliferation, degradation rate, and mechanical strength and its duration [12]. The challenge is to design a 3D scaffold constructed of natural materials that is also biocompatible with human cells. The biocompatibility of the fibers allows cells to better attach and proliferate, subsequently leading to new tissue formation and better healing and regeneration rates. To date, the main drawback related to natural scaffolding concerns its poor mechanical properties. Therefore, collagen is considered a good source of biomaterials as it allows the preservation of the original tissue shape and the ECM structure of the matrix. When collagen is subjected to the chemical processes of extraction, isolation, purification, and polymerization, its natural properties are reduced (such as the cross-linking that gives its tensile strength and proteolytic resistance to collagenase) [6]. The particular collagen source is an important parameter for consideration since at present it is derived from bovine or porcine source and thus constitutes a risk of being a vector of pathogens. Recently, it has been reported that collagen extracted from jellyfish is biocompatible and supports cell viability [16,22]. The jellyfish collagen used as a scaffold is, however, known to possess poor mechanical properties, and is thus of limited applicative use [34].

The soft-coral collagen fibers presented here have previously been studied by us at the molecular level using a proteomic analysis that enabled us to characterize their organic matrices. A molecular amino-acid sequence based on mass spectrometry (MS/MS) revealed a series of peptides [15,16] that correspond to fibrillary collagen. The sequences were analyzed, revealing that the collagen family of proteins has been well conserved through evolution, with their molecular sequence already documented in some metazoans [35]. Regarding vertebrates, 29 collagens have been characterized thus far, while for cnidarians the data available on collagens are limited (taxonomy browser NCBI—https://www.ncbi.nlm.nih.gov/protein/). Among this latter group, most of the recognized references are from sea anemones and jellyfish, and most of the reports provide only partial sequences (RefSeq Protein Database, NCBI). Moreover, the majority of collagen and collagen-like proteins described in invertebrates belong to the non-fibrillary and soluble structure subtype IV. Only a few are associated with collagen Type I, the constituent with the taxonomically closest source. A comparison of the peptides sequences obtained from the MS/MS assay on *Sarcophyton ehrenbergi* and *Sarcophyton auritum* collagen fibers to the UniProt database identify a remarkable homology to fibrillary mammalian collagen [15,16].

It is well recognized that fibrillary collagen harbors well-conserved motifs such as integrin-binding, the von Willebrand factor type A (vWFA) domain, the vWFA subfamily, the metal ion-dependent adhesion site (MIDAS) and fibronectin type 3 domain [36], and the immunoglobulin-like and cytokine receptor motif. Integrins are the main family of cell adhesion molecules, functioning to transduce signaling in and out of the cell by linking the cytoskeleton to ECM proteins, and their essential role in cell adhesion is well characterized. In addition, a laminin G domain motif recognized in the collagen fiber sequence also has a role in cell adhesion, signaling, migration, and differentiation [37]. Thus, fibrillar collagen possesses motifs that support cell adhesion and make it an essential component for scaffold formation in providing the conditions for cell attachment and spread, thereby supporting cell growth. In addition to the numerous cell adhesion motifs, high molecular weight glutenin subunits are also present in collagen fibers. This is attributed to the elastomeric proteins, which are characterized by their ability to withstand significant physical deformations without breaking, and to return to their original conformation when the stress is removed [38]. The outstanding mechanical properties of the cord-like long collagen fibers revealed in our studies [24,25,26] may thus be in part attributed to their elastomeric nature. We also identified a proteoglycan-rich organization that might underlay their structure and mechanical strength [27]. The biomolecular data on these collagen fibers isolated from *Sarcophyton ehrenbergi*, compiled from their molecular and protein features along with their mechanical properties, make these fibers a unique candidate for use as a scaffold material.

Our experiments were based on (i) cells seeded on 3% alginate (Figure 3A,C), (ii) cells seeded on collagen fibers (Figure 3B,C), and (iii) cells seeded on collagen-alginate composite (Figure 3D–F). In these cultures, we observed different cell morphologies (Figure 3) according to their substrate stiffness (Figure 2). The cells’ different morphologies were examined using live imaging, which allowed us to quantify cell shape parameters as circularity [defined as (4πArea/[Perimeter]^2)] by ImageJ software). Cells seeded on 3% alginate hydrogel displayed a circularity of 0.89 ± 0.0014 (*n* = 1222) (Figure 3A,C), while for cells seeded on collagen fibers the measured cell circularity was 0.575 ± 0.025 (*n* = 79) (Figure 3B,C), exhibiting a significant difference of *p* < 0.0001. This is explained by the stiffer properties of the collagen fibers (Figure 2), which bring to transduction of forces that lead cells to spread on the collagen fibers via adhesion molecules that bound the domains on the collagen (as described above). The transduced forces activate the cytoskeleton response to the cells’ tensional forces, demonstrating a more elongated shape. The activated signaling pathways affect cell proliferation and growth, leading to the tissue-like formation observed in Figure 3F–H.

The bio-composite was tested as a potential support for cell growth support (Figure 3D–F), by seeding cells on a collagen-alginate composite, in which the alginate hydrogel surrounded the stiffer collagen fibers. We noticed that cells sensed the different rigidities of the components, which affected their morphology (Figure 3A–C). When cells were plated on collagen fibers only they proliferated and grew for several weeks into a tissue-like structure as seen following nine weeks in culture (Figure 3G,H). This phenomenon is explained at the molecular level as tethering that underlies the cellular contractile forces, creating a connection with molecules that sense the substrate stiffness. ECM proteins anchorage and tethering play a role in regulating mechano-responsive cellular behaviors. The cellular sensing of an effective environmental rigidity combined with molecular tethering transduce through cytoskeleton backbone deformation [39]. By applying various densities of the collagen fibers in a bio-composite material, we produced a range of stiffness levels for different purposes of scaffolding. Collagen fibers able to provide different degrees of substrate stiffness, enabling different cues for cell activation, growth, and fate, can be adapted for specific applications as suggested in our previous study [7] and as related to the various applications suggested in Figure 4. The presented collagen-alginate composite, which demonstrated superior mechanical compatibility (e.g., strength and elasticity) alongside the biocompatibility properties as presented in Figure 3, therefore holds great potential for use in tissue grafts in different tissues e.g., Figure 4.

Concerning future biomedical devices, the production of soft tissue grafts that support biocompatibility and cell function together with an appropriate mechanical compatibility is still a challenge [5]. Although many efforts have been focused on the aspects of biological compatibility, the mechanical behavior of the graft is no less important. A functioning graft will require compatible mechanical properties in order to support the new tissue formation and produce the appropriate physiological activity. The challenge for researchers is thus to develop new materials for tissue repair that demonstrate biocompatibility and integration with the native tissue, while also maintaining the mechanical properties of the new tissue formed. The risk in using a mechanically unsuitable implant lies in the formation of stress concentration occurring at the interphase with the native tissue, which can lead to hyperplasia or even graft failure [5,17].

Here we used a bio-composite material that mimics the components of the natural tissue: the collagen fibers provide the load-bearing ability while the hydrogel provides the ECM-like properties for the cells’ surroundings. The effective mechanical behavior of the bio-composite lies between that of the fibers and the matrix, where the local mechanical behavior is soft enough for the cells and stiff enough to support new tissue formation, as demonstrated in the studied macroscopic mixture combined from the two constituent materials. The current study on composite materials with the potential to create a hybrid material was inspired by both industrial composites and the structure of natural tissues. Tailoring the mechanical behavior of the bio-composite to the targeted tissue can be achieved by changing the collagen fiber fraction and orientation [6,7,8].

## 3. Materials and Methods

### 3.1. Coral Used for Isolation of Collagen Fibers

*Sarcophyton* soft corals (Figure 1A) were frozen following harvesting and defrosted prior to fiber extraction. A piece of the colony was torn to expose the fibers, which were then physically pulled out from the soft coral (Figure 1B). The fibers were manually spun around a thin rectangular-shaped metal frame to create unidirectional, straight, and organized array of fiber bundles (Figure 2A). The aligned fibers were carefully washed several times in water, PBS, and then with 70% ethanol.

### 3.2. Microscopy Fiber Analysis

Fluorescence microscopy—Collagen fibers isolated from the coral were visualized under a fluorescence microscope at 305–450 nm (Optiphot, Nikon, Tokyo, Japan).

Phase contrast microscopy—Live cell cultures were observed under phase contrast microscopy and digital photography (Optiphot, Nikon, Tokyo, Japan).

Scanning electron microscopy (SEM)—For scanning electron microscopy, subsamples were fixed in 4% glutaraldehyde in filtered seawater (0.22 µm FSW), decalcified as described above, dehydrated through a graded series of ethanol up to 100%, and critical point dried with liquid CO_2_. The preparations were fractured under a compound microscope, using the tips of fine forceps, and the gastrovascular cavities were then carefully exposed. Next, they were gold-coated and examined under a scanning electron microscope (SEM Jeol-840a, Jeol LTD., Tokyo, Japan). In order to obtain fiber preparations, fibers were isolated as explained above and stored in 70% ethanol. They were then processed, coated with gold-palladium alloy, and examined at high vacuum under an environmental scanning electron microscope (SEM and ESEM, JSM-6700 Field Emission Scanning Electron Microscope, Jeol LTD., Tokyo, Japan). The diameters of collagen fibers and fibrils were obtained from the images, using the ImageJ software.

### 3.3. Bio-Composite Fabrication

The extracted fiber bundles were sterilized (Figure 2A), arranged on a metal frame, and then inserted into a dialysis membrane (6000–8000 MWCO, Spectra Por, Spectrum Labs Inc., Rancho Dominguez, CA, USA) together with 3 mL sodium alginate solution (3% *w*/*v* in DDW (Protanal LF 10/60, FMC BioPolymer, Philadelphia, PA, USA)). The membrane was sealed, flattened, and soaked in CaCl_2_ (0.02 M Ca for Tissue culture (TC) experiments or 0.1 M for mechanical testing) solution used as a cross-linker through diffusion for 48 h at room temperature to allow the gelation of the alginate to hydrogel. The bio-composite was then removed from the membrane and the frame was sterilized by immersion in 70% ethanol and further used for cell seeding. Alginate hydrogel samples were fabricated by the same protocol, excluding the embedding of fibers.

### 3.4. Fiber Volume Fraction in the Bio-Composite

The aligned fiber images were placed against a dark background and their images were acquired using a digital microscope (AM311S, BigCatch, Torrance, CA, USA). The images were processed into binary numerical arrays, and the percentage of white pixels (representing the fibers) was calculated in order to determine the fiber fraction. The fraction was normalized to the final bio-composite thickness. The calculations were done using Matlab code.

### 3.5. Mechanical Testing of the Bio-Composites and Alginate Hydrogels

Tensile testing was performed on an Instron machine model 5582 with Blue hill 2 operating software and a 100-N load cell at a rate of 0.05 mm s^−1^. The tensile measurements included three cycles of preconditioning up to 10%, followed by stretching to failure parallel to the fibers’ direction.

### 3.6. Tissue Culture

Mouse mesenchymal 3T3-L1 cells were seeded in a growth medium consisting of Dulbecco’s modified Eagle’s medium (450 mg/dL; Biological Industries, Beit Haemek Ltd., Beit Haemek, Israel), 10% fetal bovine serum (Biological Industries), 1% l-glutamine (Biological Industries), and 0.1% penicillin-streptomycin (Sigma, St. Loius, MO, USA). Cell morphology was analyzed using ImageJ software (National Institute of Health, Bethesda, MD, USA).
